# Investigation into the Reduction of Palm Oil in Foods by Blended Vegetable Oils through Response Surface Methodology and Oxidative Stability Tests

**DOI:** 10.3390/antiox13080929

**Published:** 2024-07-30

**Authors:** Vassilis Athanasiadis, Dimitrios Kalompatsios, Martha Mantiniotou, Stavros I. Lalas

**Affiliations:** Department of Food Science and Nutrition, University of Thessaly, Terma N. Temponera Street, 43100 Karditsa, Greece; dkalompatsios@uth.gr (D.K.); mmantiniotou@uth.gr (M.M.); slalas@uth.gr (S.I.L.)

**Keywords:** corn oil, rapeseed oil, fatty acids, tocopherols, volatile compounds, FT-IR, Rancimat, main effect screening design, partial least squares, consensus map

## Abstract

Recently, there has been a significant transition in the dietary preferences of consumers toward foods containing health-promoting compounds. In addition, as people’s environmental awareness increases, they are increasingly looking for sustainable solutions. Palm oil, an oil used extensively by the food industry, does not fit these criteria. This study investigated the development of a complex oil blend consisting of commonly used vegetable oils such as corn, rapeseed, sunflower, and palm oil. The aim was to find the optimal blended oil and compare this combination with palm oil in terms of its oxidative stability, antioxidant capacity, and the composition of bioactive compounds (i.e., fatty acids, tocopherols, and carotenoids). Palm oil was found to have greater oxidative stability as a result of its increased concentration of saturated fatty acids. The optimal blended oil, which consisted of corn and rapeseed oil at a ratio of 4:3 *w*/*w*, inhibited the superior antioxidant activity, showing a ~33% increase in DPPH^•^ inhibition activity. ATR-FTIR spectra further verified the existence of a significant quantity of saturated fatty acids in palm oil and unsaturated fatty acids in the blended oil. Finally, several correlation analyses revealed interesting connections between oil samples and investigated parameters. This work has the potential to establish a basis for the mass production of oil blends that possess high concentrations of antioxidant compounds and reduce the use of palm oil.

## 1. Introduction

Palm oil, despite its widespread use in food and cosmetic manufacturing, has faced allegations in recent years over its detrimental effects on health, mostly due to its elevated levels of saturated fatty acids (SFAs) [1,2,3]. Additionally, it has been associated with environmental degradation, including deforestation and the depletion of biodiversity [4]. The primary objection to the consumption of palm oil is its high content of palmitic acid, which is a SFA [1]. Crude palm oil may contain amounts of peroxides, chromophores, metals, and other pollutants that have the potential to impact human health. Palm oil requires a refining process (i.e., degumming, refinement, bleaching, and deodorization) to overcome this limitation and become edible [5]. Moreover, in the past few years, the growing awareness among consumers regarding a healthy diet has caused a debate regarding the consumption of palm oil [3]. Regulations and public awareness could reduce the use of palm oil in food sectors in Europe [6]. While completely replacing palm oil in the food sector may not be feasible yet, there is ample opportunity for the introduction of alternative vegetable oils that are versatile, nutritious, environmentally friendly, and economically viable [7].

Vegetable oils consist of triacylglycerides combined with SFAs and unsaturated fatty acids (UFAs), phospholipids, pigments, phytosterols, and tocopherols. Vegetable oils are among the most important and industrially used substances for fortifying foods and producing functional foods, due to their high content of ω-3 and ω-6 fatty acids [8]. The latter plays an important role in enhancing human health but cannot be produced by the human body. Obtaining them from food is hence of utmost importance. Cooking oil is the most prevalent source of ω-3 and ω-6 fatty acids [8]. Sunflower seeds are considered one of the most cultivated oilseeds worldwide, along with rapeseed, soybean, and cottonseed [9,10]. Oilseeds usually serve as a primary source of vegetable oils that possess distinctive physicochemical characteristics [9]. Rapeseed is a highly prevalent edible oil worldwide, ranking second only to soybean oil in terms of production [11]. The introduction of novel rapeseed cultivars with reduced levels of erucic acid and glucosinolate has garnered worldwide interest in utilizing rapeseed as a valuable source of edible oil. Canola oil refers to the oil derived from rapeseed types that have low levels of erucic acid (<2%) and glucosinolates [11].

The balance between SFAs and UFAs serves a crucial role in human nutrition. SFAs, despite their role in oil stability, have been associated with the development of cardiovascular disease [12]. Vegetable oils that are rich in UFAs are currently being heavily recommended due to their potential health benefits. These types of fatty acids have been found to help reduce low-density lipoproteins, prevent clotting, and inhibit the proliferation of vascular smooth muscle [13]. This could be attributed to the presence of double bonds in these molecules; however, they are more susceptible to oxidation. The exposure of oils to light, heat, and oxygen accelerates the oxidation of fatty acids, leading to a decline in the nutritional quality of the oils [14]. The most typical way to process most of the products in the market today is by frying them. The elevated temperature and exposure to oxygen during the frying process leads to oxidation, polymerization, degradation, and hydrolysis. These reactions generate several nutritionally detrimental compounds that have a negative effect on the quality of both the oil and the fried food [15].

Recently, there has been a rising demand for food that possesses beneficial properties for consumers, alongside a growing consciousness of the environment. In addition, people have begun to utilize vegetable oils as a viable substitute for olive oil due to their bioactive compounds content. This research aims to investigate the oxidative stability of vegetable oil blends, as well as their fatty acid and tocopherol content. The vegetable oils under investigation include corn oil, sunflower oil, rapeseed oil, and palm oil. The ultimate goal is to explore the potential for completely or partially replacing palm oil with these blends. The findings of this study may suggest an appropriate combination of oils that possess a high level of nutritional quality, which might be extensively employed in the food industry.

## 2. Materials and Methods

### 2.1. Chemicals and Reagents

Malondialdehyde and tocopherol standards were bought from Merck Ltd. (Darmstadt, Germany). Thiobarbituric acid (TBA), trichloroacetic acid, hydrochloric acid (37%), acetone, glacial acetic acid, and ammonium iron (II) sulfate were all obtained from Panreac (Barcelona, Spain). Trolox (6-hydroxy-2,5,7,8-tetramethylchroman-2-carboxylic acid) was bought from Glentham Life Sciences (Corsham, UK). Isooctane, ethyl acetate, and dichloromethane were purchased from Carlo Erba (Vaul de Reuil, France). Chloroform, sodium chloride, and ammonium thiocyanate were purchased from Penta (Prague, Czech Republic). From Sigma Aldrich (St. Louis, MA, USA), *p*-anisidine, 2-propanol, 2-octanol, and cyclohexane were purchased. Hydrogen peroxide (35%) was bought from Chemco (Malsch, Germany). High-purity ethanol (99.8%) was obtained from Fischer Scientific (Loughborough, UK). FAME Mix C_8_–C_24_ reference standards were purchased from Sigma-Aldrich, St. Louis, MO, USA. Deionized water was generated from a deionizing column.

### 2.2. Materials

Fresh refined palm, sunflower, corn, and rapeseed oils were obtained from a local market in Karditsa city (Greece). All oil samples were stored in the refrigerator at a temperature <4 °C. To achieve thermal equilibrium between them and room temperature, they were kept in the dark (20 °C) in a temperature-controlled room and were immediately analyzed after opening.

### 2.3. Instrumentation

Accelerated oil oxidation was done through a Metrohm LTD Rancimat 743 apparatus (Herisau, Switzerland). A Shimadzu UV-1900i UV/Vis spectrophotometer (Kyoto, Japan) was used for spectrophotometric analyses. For the chromatographic determination of tocopherols, a Shimadzu CBM-20A (Shimadzu Europa GmbH, Duisburg, Germany) high-performance liquid chromatography (HPLC) apparatus equipped with a SIL-20AC autosampler and a CTO-20AC column oven were used. Specifically, a Shimadzu RF-10AXL fluorescence detector was employed, with a column (125 Å, 10 μm, 3.9 mm × 300 mm) from Waters *μ*-Porasil (Milford, MA, USA). A gas chromatograph with a flame ionization detector (GC-FID) from Agilent Technologies (Santa Clara, CA, USA) Gas Chromatograph model 7890A, equipped with a capillary column Omegawax (30 m × 320 μm × 0.25 μm) from Supelco (Bellefonte, PA, USA), was used to quantify fatty acids. A divinylbenzene/carboxene/polydimethylsiloxane (DVB/CAR/PDMS) coating SPME fiber from Supelco (Bellefonte, PA, USA) was employed to absorb volatile compounds, whereas an Agilent Technologies (Santa Clara, CA, USA) Gas Chromatograph model 7890A linked to a mass selective detector model 5975C, equipped with an Agilent J&W DB-1 capillary column (30 m × 320 μm × 0.25 μm) (Folsom, CA, USA), was used to identify and quantify the odorous volatile secondary oxidation by-products. To identify the chemical composition of oil samples, an Attenuated Total Reflectance (ATR) apparatus with a ZnSe crystal trough plate was used to acquire the Fourier-Transform Infrared (FTIR) spectra from a Prestige21 spectrophotometer purchased from Shimadzu (Kyoto, Japan). The apparatus also included a ceramic light source, a KBr beam splitter, high-energy throughput optical elements, and a deuterated triglycine sulphate doped with *L*-alanine.

### 2.4. Vegetable Oil Blend Optimization

The vegetable oils were blended in a total sum of 100 g in different ratios (vide infra) with the Main Effects Screening design shown in Table 1. Each vegetable oil was denoted with variables *X*_1_−*X*_4_, as well as their respective ratios in the Coded Variable Levels. Response surface methodology (RSM) was used to find both the largest peak area and the impact of a significant independent variable on the response. Both the overall model significance (R^2^, *p*-value) and the significance of model (equation) coefficients were verified at a minimum level of 95% using an analysis of variance (ANOVA) and lack-of-fit tests.

Furthermore, as Equation (1) demonstrates, the response variable was predicted as a function of the examined independent components using a second-order polynomial model:

(1) $$Y_{k}=\beta_{0}+\sum_{i=1}^{2}\beta_{i}X_{i}+\sum_{i=1}^{2}\beta_{ii}X_{i}^{2}+\sum_{i=1}^{2}\sum_{j=i+1}^{3}\beta_{ij}X_{i}X_{j}$$

where *β*_0_, *β*_i_, *β*_ii_, and *β*_ij_ are the intercept and regression coefficients of the linear, quadratic, and interaction terms of the model, respectively; *Y*_k_ is the predicted response variable; and *X*_i_ and *X*_j_ are the independent variables.

### 2.5. In Vitro Antioxidant Activity Through Radical Inhibition

The inhibition of 2,2-diphenyl-1-picrylhydrazyl radical (DPPH^•^) was measured with an established method from Kalantzakis et al. [16] with some modifications, since the authors used 1 mL of 10% *w*/*v* diluted oil and 4 mL of freshly prepared 100 μM DPPH^•^ solution in ethyl acetate. In brief, a volume of 950 μL of DPPH^•^ solution (100 μM in ethyl acetate) was mixed with 50 μL of a 10-fold diluted oil sample. The absorbance at 515 nm was immediately recorded spectrophotometrically with a blank sample (*A*_515(i)_), whereas the absorbance of the oil sample was measured after 30 min (*A*_515(f)_). The inhibition of DPPH^•^ was calculated as shown:

(2) $$\mathrm{Inhibition}\left(\%\right)=\left(\frac{A_{515(i)}-A_{515(f)}}{A_{515(i)}}\right)\;\times \;100$$

The results were expressed as μM Trolox equivalent antioxidant capacity (TEAC) per kg of oil using a calibration curve (y = 0.1566x + 0.6698, R^2^ = 0.999) which was prepared with Trolox (50–500 μM).

Equation (3) below was used to determine the percentage of the oxidative stability of oils (%OS) in DPPH inhibition both before and after a 2-h incubation in boiling water:

(3) $$\mathrm{Oxidative}\;\mathrm{Stability}\;\left(\%\right)=1-\left(\frac{{\mathrm{DPPH}\;\mathrm{inhibition}}_{\;2\;h}-{\mathrm{DPPH}\;\mathrm{inh}\mathrm{ibition}}_{\;0\;h}}{{\mathrm{DPPH}\;\mathrm{value}}_{\;0\;h}}\right)\;\times \;100$$

### 2.6. Bioactive Compounds Determination

#### 2.6.1. Tocopherol Quantification

The tocopherol quantification was done using a previously established method by Lalas et al. [17]. In brief, an amount of 0.25 g of oil was precisely weighed into a 5 mL volumetric flask, diluted with *n*-hexane, and vigorously shaken. A volume of 20 μL of an oil sample was directly injected into the HPLC apparatus. The mobile phase included *n*-hexane/2-propanol/absolute ethanol (97.5:2.0:0.5, *v*/*v*/*v*) at a flow rate of 1 mL/min. The fluorescence detector was set at 294 nm for excitation and 329 nm for emission. The tocopherol content (TC) was expressed as mg of each tocopherol per kg of oil, using the following equation:

(4) $$\mathrm{TC}\;(\mathrm{mg}\;T/\mathrm{kg}\;\mathrm{Oil})=\frac{C_{T}\;\times \;V\;\times \;1000}{w}$$

where *C*_T_, *V*, and *w* denote the concentration of each tocopherol (i.e., α-, β-, γ-, and δ-) (in mg/L), the volume of the extraction medium (in L), and the weight of the oil sample (in g), respectively.

#### 2.6.2. Total Carotenoid Content (TCC)

The total carotenoids were calculated using the method described by Minguez-Mosquera et al. [18] and were expressed as mg of lutein per kg of oil. A solution of 5 mL of cyclohexane and 1.5 g of oil was prepared. The absorbance was immediately recorded spectrophotometrically at 470 nm. For lutein, it was previously documented [18,19] that the extinction coefficients (*E*_0_) that were used had a value of 2000 M^−1^ cm^−1^ [18]. Calculations for TCC were performed as follows by using the same equation as Borello and Domenici [19]:

(5) $$\mathrm{TCC}\;(\mathrm{mg}/\mathrm{kg})\;=\frac{A_{470}\;\times \;10^{6}}{E_{0}\;\times \;100\;\times \;d}$$

where spectrophotometer cell thickness is denoted as *d* (1 cm).

#### 2.6.3. Fatty Acids Composition

The method from Commission Regulation (EC) No 796/2002 (Annex XB) [20] was used for the preparation of the fatty acid methyl esters (FAMEs) from oils, whereas the methyl esters quantification was done using a GC-FID method previously described by Lalas et al. [21]. The column temperature program was determined to be isothermic for 5 min at 70 °C, ramped up to 160 °C at a rate of 20 °C/min, then raised to 200 °C at a rate of 4 °C/min, and raised to 240 °C at a rate of 5 °C/min. Temperatures for the injector and FID were kept at 240 and 250 °C, respectively. At a flow rate of 1.4 mL/min (i.e., 27 cm/sec velocity), helium served as the carrier gas. Hydrogen, air, and helium flows were set at 50, 450, and 50 mL/min. Split mode (1:100) was used to inject 1.0 μL samples. A comparison of the individual peaks with FAME Mix C_8_–C_24_ reference standards allowed for the identification. By using the normalization method (without correction factors), the percentage composition of the samples was determined from the GC peak areas. The component percentages were computed as averages of three GC-FID analyses.

In addition, the calculated oxidizability value (COX) from fatty acids was determined as per previous studies [22,23] by using the method initially proposed by Fatemi and Hammond [24], as indicated in Equation (6):

(6) $$\mathrm{COX}=\frac{1\;\left(18:1,\;\%\right)+10.3\;\left(18:2,\;\%\right)+21.6\;(18:3,\;\%)}{100}$$

To determine the atherogenicity index (IA) and thrombogenicity index (IT), the formulae provided by Ulbricht and Southgate [25] were used:

(7) $$\mathrm{IA}=\frac{\left[C12:0+\left(4\;\times \;C14:0\right)+C16:0\right]}{\sum \mathrm{UFA}}$$

(8) $$\mathrm{IT}=\frac{\left(C14:0+C16:0+C18:0\right)}{[(0.5\;\times \sum \mathrm{MUFA})+(0.5\;\times \sum \omega \text{-}6)+(3\;\times \sum \omega \text{-}3)+(\omega \text{-}3/\omega \text{-}6)]}$$

The calculation of the hypocholesterolemic/hypercholesterolemic (HH) ratio was based on the work of Santos et al. [26], as follows:

(9) $$\mathrm{HH}=\frac{(C18:1+\sum \mathrm{PUFA})}{\left(C12:0+C14:0+C16:0\right)}$$

Finally, to evaluate the nutritional quality of dietary fat, Chen et al. [27] suggested the health-promoting index (HPI), which is centered on the impact of fatty acid composition on cardiovascular diseases, as per Equation (10):

(10) $$\mathrm{HPI}=\frac{\sum \mathrm{UFA}}{\left[C12:0\;+\left(4\;\times \;C14:0\right)+C16:0\right]}$$

### 2.7. Accelerated Oil Oxidation Process

The oxidative stability with an accelerated oxidation process was determined using a previously described method [28]. For the full oxidation of oils, the Rancimat device was set up to 100−130 °C and 15 L/h of airflow, and the mass of the oil sample used was 3 ± 0.01 g. Based on the plotted graph, the onset of oxidative degradation, or rancidity, was identified as the point of abrupt conductivity increase in measuring cells that hold 60 mL of deionized water. 873 Biodiesel Rancimat software (version 1.00) was linked to the device.

### 2.8. Oxidation By-Product Measurement

#### 2.8.1. Peroxide Value (PV) Assay

The peroxide values of all oil samples were determined using a modified version of the IDF standard method 74A:1991 [29]. The original method relies on the co-oxidation of Fe^+2^ to Fe^+3^ by hydroperoxides and the subsequent formation of the reddish Fe^+3^-thiocyanate complex. Specifically, it demanded the use of 0.33 g of oil sample mixed with 9.6 mL of a solvent consisting of methanol, 1-decanol, and *n*-hexane, 0.05 mL of ammonium thiocyanate solution, and 0.05 mL of iron chloride. Briefly, in a 2 mL Eppendorf tube, 2 mL of dichloromethane/ethanol solvent (3:2, *v*/*v*) was mixed with 0.05 g of oil sample and vortexed for 2–4 s. A quantity of 20 μL of oil sample extract was combined with 1960 μL of solvent. After adding 10 μL of a 4 M ammonium thiocyanate solution in water and 10 μL of ammonium iron (II) sulfate solution (25.5 mM in 10 M HCl), it was swiftly mixed with vortex. The absorbance of the sample was measured spectrophotometrically at 500 nm against a blank solution (i.e., reaction mixture without oil sample) after 5 min storage at room temperature in the absence of light. Using this process, a hydrogen peroxide (H_2_O_2_) calibration curve with six different concentrations (50–500 μmol/L in DCM/EtOH, y = 0.0004x + 0.0524, and R^2^ = 0.9950) was built and the PV was calculated. The PV was computed using the following formula and expressed as mmol H_2_O_2_ per kg of oil:

(11) $$\mathrm{PV}\;\left(\mathrm{mmo}l\;H_{2}O_{2}/\mathrm{kg}\;\mathrm{Oil}\right)=\frac{C_{H_{2}O_{2}}\;\times \;V}{w}$$

where $C_{H_{2}O_{2}}$ denotes the H_2_O_2_ concentration (in μmol/L); *V* defines the volume of the extraction medium (in L), and *w* is the weight of the reaction oil sample (in g).

#### 2.8.2. Conjugated Dienes and Trienes Determination

The procedure for determining the values of conjugated dienes and trienes was performed as described by Pegg et al. [30]. Cyclohexane was added to a 5 mL volumetric flask along with 0.01 g of the oil sample. Conjugated dienes and trienes had absorbances measured at 232 and 270 nm, respectively.

The values of conjugated dienes (CD) and trienes (CT) were determined by employing the following equations by Pegg et al. [30]:

(12) $$C_{\mathrm{CD}}\;(\mathrm{mmol}/\mathrm{mL})\;=\frac{A_{232}}{\varepsilon \;\times \;l}\;\;\;\;\;\;\;\;\;\;{\mathrm{CD}}_{\mathrm{value}}\;(\mathrm{mmol}/\mathrm{kg}\;\mathrm{Oil})=\frac{C_{\mathrm{CD}}\;\times \;(5\;\times \;10^{3})}{w}$$

(13) $$C_{\mathrm{CT}}(\mathrm{mmol}/\mathrm{mL})\;=\frac{A_{270}}{\varepsilon \;\times \;l}\;\;\;\;\;\;\;\;\;\;\;{\mathrm{CT}}_{\mathrm{value}}\;(\mathrm{mmol}/\mathrm{kg}\;\mathrm{Oil})=\frac{C_{\mathrm{CT}}\;\times \;(5\;\times \;10^{3})}{w}$$

The molar absorptivity (*ε*) of linoleic acid hydroperoxide is 2.525 × 10^4^ M^−1^ cm^−1^, as previously documented [30], whereas *l* is the path length of the cuvette in cm (1 cm), $C_{\mathrm{CD}}$ and $C_{\mathrm{CT}}$ are the CD and CT concentration in M (the molar concentration); *A*_232_ and *A*_270_ are the absorbances of the lipid solution at 232 nm and 270 nm; and 5 × 10^3^ is a factor that includes the volume of solvent (5 mL) used to dissolve the oil sample. The content of CD and CT can be expressed in mmol per kg of oil.

Equation (14) below was used to determine the percentage of the oxidative power (%OP) of oils in CD and CT values both before and after the 2-h incubation in boiling water:

(14) $$\mathrm{Oxidative}\;\mathrm{Power}\;\left(\%\right)=\left(\frac{{\mathrm{CD},\mathrm{CT}\;\mathrm{value}}_{\;2\;h}-{\mathrm{CD},\mathrm{CT}\;\mathrm{value}}_{\;0\;h}}{{\mathrm{CD},\mathrm{CT}\;\mathrm{value}}_{\;0\;h}}\right)\;\times \;100$$

#### 2.8.3. Thiobarbituric Acid Reactive Substances (TBARSs) Assay

The TBARSs assay was conducted according to the methodology defined by Qiu et al. [31]. A volume of 5 mL of TBA solution and 0.1 g of oil sample were added to a tube. The mixture was shaken well and left for 20 min at 95 °C. The TBA solution required 15 g of trichloroacetic acid, 1.76 mL of concentrated HCl, and 0.375 g of TBA in a 100 mL volumetric flask filled with deionized water. The samples were left for 5 min in an ice bath following incubation. The mixture was vortexed after 200 μL of chloroform was added, and then centrifuged for 10 min at 4500 rpm. With the UV spectrophotometer set at 532 nm, the absorbance of the supernatant was determined. Deionized water was used to replace the sample in the preparation of a blank solution. Using a malondialdehyde calibration curve (15–300 mmol/L in deionized water, y = 0.0032x–0.0004, R^2^ = 0.9999) and the following equation, the TBA value was determined as mmol of malondialdehyde equivalents (mmol MDAE) per kg of oil:

(15) $${\mathrm{TBA}}_{\mathrm{value}}\;(\mathrm{mmol}\;\mathrm{MDAE}/\mathrm{kg}\;\mathrm{Oil})=\frac{C_{\mathrm{MDA}}\;\times \;V}{w}$$

where *w* is the weight of the oil sample (in g); *V* is the volume of the extraction medium (in L); and *C*_MDA_ is the concentration of malondialdehyde (in mmol/L).

#### 2.8.4. Volatile Compounds Determination by HS-SPME/GC-MS

A slightly modified version of a previously published methodology by [32] was utilized, which refers to the headspace solid-phase microextraction (HS-SPME) methodology. The fiber was pre-conditioned for 30 min at 270 °C, as per the manufacturer’s recommendation. To conduct the HS-SPME extraction, a 25 mL glass vial was filled with 10 mL of the water from the Rancimat apparatus sample (i.e., which contained volatile oxidation by-products), 3 g of sodium chloride, and 2 mg/L of 2-octanol, which served as an internal standard. A PTFE/silicone septum was subsequently used to firmly close the vial. The vial was maintained in a water bath at 40 °C for the entire duration of the extraction (40 min) and equilibration (10 min). In the vial head area, the fiber was positioned above the water surface. All experiments were conducted with constant stirring at 500 rpm. After the extraction was complete, the fiber was removed from the vial, threaded through the needle, and placed into the injector of the gas chromatograph.

A method that has been described elsewhere was modified for the purpose of conducting the GC-MS analysis [32]. To start with, the flow rate of the helium carrier gas was 1.5 mL/min. Operating in splitless mode, the injector reached a temperature of 240 °C. The column was preheated to 40 °C for 5 min, and then gradually raised to 140 °C at a rate of 2 °C/min. Lastly, it was heated to 240 °C for 10 min at a rate of 10 °C/min. In total, the runtime was 75 min. The MSD parameters demanded mass range *m*/*z* 29–350; source temperature 230 °C; quadrupole temperature 150 °C; and acquisition mode electron impact (EI) 69.9 eV. The results were compared with the EI mass spectra libraries NIST11 (NIST, Gaithersburg, MD, USA) and W8N08 (John Wiley & Sons, Inc., Hoboken, NJ, USA), whereas Agilent Technologies MSD Chemstation software (version E.02.00.493) was employed to evaluate all of the chromatogram peaks/spectra. The GC peak regions (without correction factors) were used to determine the sample composition using the normalization approach. To evaluate the quantities of volatile compounds, the average data from multiple GC-MS analyses were utilized. These amounts were subsequently expressed as micrograms of 2-octanol equivalents per kg of oil.

#### 2.8.5. *p*-Anisidine Value (*p*-AV) Assay

The *p*-Anisidine value was calculated according to ES ISO 6885:2012 [33]. A 10 mL volumetric flask contained 0.5 g of oil sample and 10 mL of isooctane in total. A volume of 1 mL of the dilute oil sample solution was inserted into a tube, and mixed with 200 μL of glacial acetic acid, with the absorbance *A*_0_ being measured. Furthermore, 1 mL of the diluted oil sample solution was added to 200 μL of *p*-anisidine solution (0.5% *w*/*v* in glacial acetic acid) and shaken well, and the corresponding absorbance (*A*_1_) was measured. Finally, 1 mL of isooctane was added to 200 μL of *p*-anisidine solution, shaken well, and absorbance (*A*_2_) was measured. All absorbances were measured at 350 nm and after keeping each sample in the absence of light for 10 min. Calculating *p*-AV required the following equation:

(16) $$p\text{-}\mathrm{AV}=\frac{100\;Q\;V}{m}\;0.24[(A_{1}-A_{2}-A_{0})]=12(\frac{A_{1}-A_{2}-A_{0}}{m})$$

where *m* is the mass of the test portion (~0.5 g), *V* denotes the volume (10 mL), and *Q* is the sample content of the measured solution (0.05 g/mL). The absorbance of the unreacted test solution is denoted by *A*_0_; that of the reacted solution by *A*_1_; and that of the blank by *A*_2_. The correction factor for 200 μL of the reagent or glacial acetic acid (+20%) dilution of the test solution is 0.24.

#### 2.8.6. Assessment of Total Oxidation By-Products

A measure of overall oxidation, including products of primary and secondary oxidation, is the Totox value (TV). The Totox value was determined using the established method by Sun-Waterhouse et al. [34], as follows:

TV = 2 × PV + *p*-AV (17)

where PV refers to the peroxide value as mmol H_2_O_2_ per kg of oil and *p*-AV refers to the *p*-anisidine value.

### 2.9. ATR–FTIR Qualitative Analysis

The chemical composition of each oil sample was performed by using 0.8 mL and 32 scans at a resolution of 4 cm^−1^, covering a range of 4000–400 cm^−1^. The ATR crystal was wiped down with an acetone-soaked tissue both before and after the measurement to remove any trace of oil. By employing Shimadzu IRsolution (version 1.60), the FTIR spectra of every sample was processed. Sample spectra were recorded for baseline and signal-to-noise ratio analysis.

### 2.10. Statistical Analysis

Triplicate determinations were used in the analyses; thus, the results are shown as such. A one-way analysis of variance (ANOVA) test was used to determine the statistical significance of the variances between mean values; *p* < 0.05 was regarded as statistically significant. JMP™ Pro 16 software (SAS, Cary, NC, USA) was utilized to execute the experimental design for the RSM and all corresponding statistics. All correlation analyses (i.e., partial least squares analysis, multiple factor analysis, consensus map, and multivariate correlation analysis) were conducted with the same software.

## 3. Results and Discussion

The purpose of this research was to find an oil blend with high nutritional quality that could potentially reduce the global use of palm oil (PO), which has high thermal oxidative stability [35]. Along with palm oil, the three most common oils produced in the EU (i.e., corn oil, rapeseed oil, and sunflower oil) were selected for this purpose [36]. On top of that, no added chemical antioxidants were listed on the labels. The design point samples were initially incubated in boiling water for two hours. To determine the optimal oil blend, the antioxidant activity was assessed by DPPH^•^ inhibition, and the formation of conjugated dienes and trienes was evaluated as part of the screening process. Afterward, the optimal blended oil (OBO) sample was generated through partial least squares analysis and was assessed in comparison to the PO sample using various methods to measure bioactive compounds, such as tocopherols, carotenoids, and fatty acids. Primary and secondary oxidation by-products were measured spectrophotometrically, whereas specific odorous volatile compounds were identified and quantified using the HS-SPME/GC-MS method. ATR-FTIR analysis was employed to thoroughly investigate the chemical composition of the samples. Correlation analyses were also conducted to investigate any possible correlation between all mentioned variables.

### 3.1. Alterations in Antiradical Activity and Oxidative Stability in Oil Blends

Potential physical and chemical interactions between oil components, as well as the combined effect of a diverse array of antioxidant components against oxidation processes in these oils, are incorporated into the total antioxidant capacity of edible vegetable oils. Thus, attributes such as the total antioxidant capacity, the bioactivity, and the oxidative stability of edible oils may be used as quality markers [37]. The inherent capacity of each oil to scavenge radicals could be caused by their naturally occurring lipophilic antioxidants or their fatty acid composition [38]. To identify the variations in the oxidative state that could affect their capacity to scavenge radicals, the Trolox-equivalent antiradical activity (TEAC) and conjugated fatty acid concentration of the samples were measured both at the start and end of the two-hour incubation in boiling water. Additional information is presented in Table 2. Corn oil exhibited the highest initial DPPH value, measuring 243.76 μmol TEAC/kg oil, whereas palm oil demonstrated the lowest antioxidant activity, measuring 162.66 μmol TEAC/kg oil. It is nevertheless pertinent to note that although design point 3 samples initially showed low antioxidant activity, they ultimately displayed the highest percentage of oxidative stability (91.32%). This finding indicated that it was the most resistant oil sample after being subjected to boiling water for two hours. Regarding conjugated dienes and trienes, the values between conjugate dienes ranged from 7.13–16.33 and 1.89–8.59 mmol/kg oil, respectively. The resistance against oxidation seems to be significantly influenced by the composition of the oils in the mixture. For instance, design point 2 was rich in sunflower oil and had the highest conjugated dienes value. On the other hand, design point 11 had the lowest value and was mostly composed of rapeseed and corn oil and was poor in sunflower oil. However, design point 2 had a strong oxidative power of 0.86% compared to other oils. The values in conjugated dienes after two hours of incubation in boiling water varied from 12.24–39.72 mmol/kg oil. Having mostly corn and rapeseed oils in its composition, design point 14 had the lowest value. A similar trend was observed in conjugated trienes values in both measurements before (1.89–8.59 mmol/kg oil) and after incubation (3.72–9.56 mmol/kg oil). It was again proved that the presence of sunflower oil-rich blended oils had a negative effect on their oxidative stability and antioxidant activity. For example, design point 7 had the lowest oxidative stability and oxidative power of all samples. Eventually, it could be concluded that rapeseed oil has low oxidative resistance as a substrate oil but was effective against oxidation effects when used in an oil blend. In a study by El-Reffaei et al. [39], the contribution of rapeseed, sunflower, and palm olein oil blends to oxidative stability was studied. It was shown that the sunflower and rapeseed oil blend not only represented a healthier oil blend in terms of health-protecting compounds but also reduced the rate of the conjugated dienes value in multiple deep-frying cycles compared to pure rapeseed oil.

### 3.2. Blended Vegetable Oil Composition Optimization

**Figure 1:**
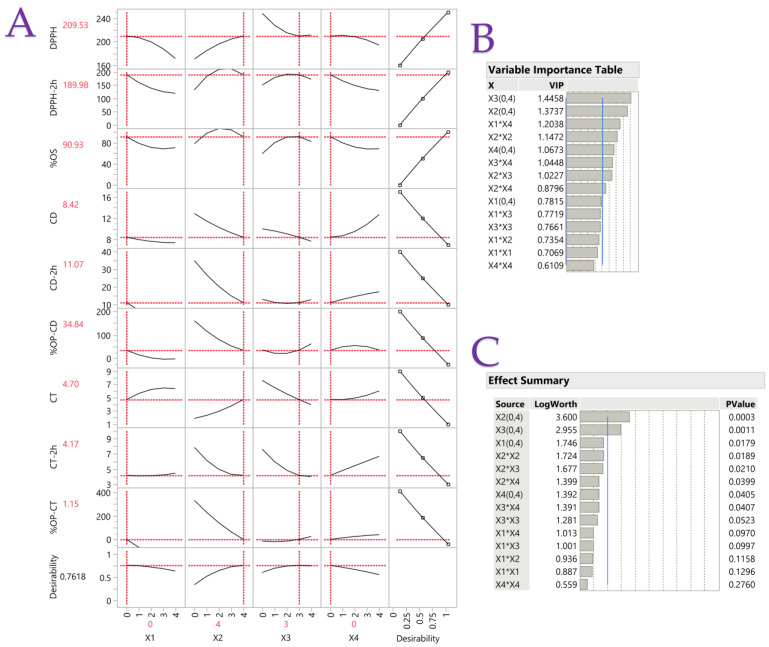
Plot (**A**) displays the desirability function and PLS prediction profiler for each variable when optimizing blended vegetable oils. The plot (**B**) table displays the VIP values for each predictor variable on the Variable Importance Plot (VIP) option graph. The VIT at 0.8 shows a blue dashed line representing each variable’s significance level. Additionally, the Effect Summary table is shown in plot (**C**) with a blue reference line at two and dashed vertical lines at integer values. At the 0.01 level, a value greater than two is significant because −log10(0.01) = 2.

After analyzing the oil samples, certain deductions were made about the capacity to evaluate the antioxidant activity and oxidative stability (conjugated dienes and trienes). The utilization of partial least squares (PLS) analysis facilitated the identification of the optimal oil composition for the blend, which effectively balanced both oxidative stability and high antioxidant activity. By doing so, we would acquire a blended oil of exceptional quality that is appropriate for both the frying process and human consumption, ultimately safeguarding human health through bioactive compounds. Figure 1A illustrates the impact of each parameter in oil samples. It was first noted that while the recommended blend should have substantial amounts of corn and rapeseed oil, it should not contain any palm or sunflower oil. A similar trend was observed in conjugated diene and triene values. Figure 1B showed the positive impact of each variable (combined or not) on the final blend, whereas Figure 1C revealed that only *X*_2_ and *X*_3_ variables had the most statistically significant (*p* < 0.01) impact. In both latter figures, Coded Units are shown with their minimum and maximum Coded Variable Levels (i.e., *X*_3_ (0,4)). SFAs abound in palm oil, but stability tests revealed sunflower oil to be inadequate. Considering this, the most suitable oil combination involves the total absence of both oils and recommends the use of three parts rapeseed oil and four parts corn oil.

**Figure 2:**
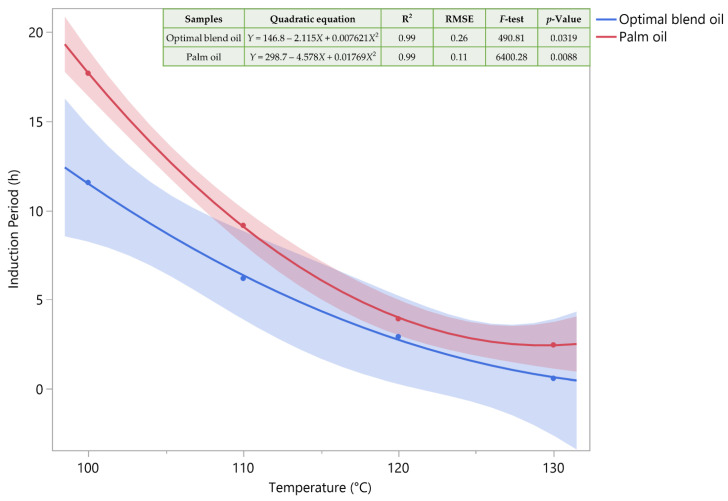
Using a polynomial regression fit, the induction period and temperature are related. A few statistics are included in the inset table.

As such, OBO was subjected to the same procedures as PO and compared (vide infra). Figure 2 illustrates, through a polynomial regression fit, the connection between temperature and the induction period by Rancimat apparatus. To fully understand the kinetics under heat exposure required this graphical representation. The curve that results from the polynomial regression gives important information for predictive modeling and analysis on the rate at which the induction period varies with temperature. Accelerated oxidation at 100–130 °C showed that palm oil is more resistant to oxidation. This result was expected since polyunsaturated fatty acids (PUFAs) are more susceptible to oxidation than SFAs [40,41]. However, they exhibit a comparable pattern at temperatures higher than 130 °C, which is more proof that their stability resembles those under this type of exposure.

### 3.3. Bioactive Compounds Quantification and Antioxidant Capacity of OBO and PO Samples

#### 3.3.1. Comparison of Total Carotenoids, Total Tocopherols, and Antioxidant Activity

The degradation of the bioactive compounds (i.e., total carotenoids and total tocopherols) of OBO, as well as the assessment of its antioxidant capacity through DPPH^•^ binding, were compared to palm oil when both oils were exposed to accelerated oxidation conditions using the Rancimat apparatus at 110 °C at 15 L/h for up to 4 h of accelerated oxidation. The results, which are shown in Table 3, revealed that the pure substrate oils were initially shown to have a greater antioxidant capacity than PO and OBO. Corn and rapeseed oils had the highest values at 245.59 and 171.56 μmol TEAC/kg oil, respectively. The combination of the two oils (i.e., OBO sample) was found to have a lower antioxidant capacity than the two oils (149.08 μmol TEAC/kg oil). However, OBO had better antioxidant activity than PO (111.92 μm TEAC/kg oil). In addition, both oils were found to have lost ~50% of their antioxidant activity after 4 h of the accelerated oxidation process, with OBO having ~2.5-fold higher antioxidant capacity than PO (73.55 μm TEAC/kg oil).

Most seed oils consist of several bioactive compounds that safeguard human health due to their antioxidant activity. Eye diseases and several types of cancer (i.e., liver, breast, and prostate) could be prevented by consuming foods rich in carotenoids. Most of the health advantages of carotenoids may be attributed to their antioxidant properties [42]. The carotenoid content from the two samples revealed a ~30% reduction from 2.80 to 1.95 mg lutein/kg oil in OBO after 4 h of accelerated oxidation process, whereas ~46% reduction was measured in PO. It was revealed that OBO sample had significantly higher carotenoid content than PO, both before and after an accelerated oxidation process. Another major type of bioactive compound, tocopherols are hydrophobic chemical compounds that consist of α-, β-, γ-, and δ- homologs and constitute the vitamin E group. There is evidence that tocopherols, the main class of lipid-soluble antioxidants, may prevent cardiovascular disease and several malignancies. Additionally, tocopherols are critical in halting the autoxidation chain process by capturing the hydroperoxide intermediates [43]. Tocopherol antioxidant activity is due to their capacity to inhibit lipid peroxidation by donating their phenolic hydrogen to radicals that do not contain any lipids [44]. Regarding tocopherol content in oil samples, a similar pattern to the DPPH^•^ value was observed, wherein the OBO sample had a total tocopherol value between that of the two pure oils (691.34 mg/kg oil). This content was found to be significantly higher than PO (i.e., 526.98 mg/kg oil), in which β-tocopherol was not quantified. The total tocopherol composition of PO and OBO was investigated under accelerated oxidation conditions for up to 4 h. The research revealed that the reduction of tocopherols in the OBO sample was less than that in the PO sample (~50% compared to ~75%, respectively), suggesting a greater stability in the specific compounds. A similar study was conducted by Mba et al. [45] who investigated the stability of virgin palm oil, refined rapeseed oil, and their 1:1 *w*/*w* blend. The initial tocopherol content of PO (456.16 mg/kg), rapeseed oil (182.44 mg/kg), and their blend (272.99 mg/kg) was measured. The deterioration of total tocopherols during exposure to high temperature was greater in PO, following a similar trend to our study.

#### 3.3.2. Fatty Acids Composition and Indices

A variation in the fatty acids composition has a crucial role in assessing the oil quality, as it is directly linked to the level of oxidation [14]. Table 4 displays the fatty acid composition of PO and OBO at various stages of oxidation, along with the initial composition of corn and rapeseed oils. It is evident that the fatty acid composition in both OBO and PO has remained relatively stable during oxidation. Nevertheless, the OBO contains a higher concentration of PUFAs, while the monosaturated fatty acids (MUFAs) showed no statistically significant difference (*p* > 0.05) between the two oils. There was minimal change in the MUFA:PUFA ratio over time in both oils. Due to this factor, OBO contains nearly double the amount of UFAs, which explains why OBO is very susceptible to oxidation. It is important to mention that the percentage of palmitic acid in PO is approximately 44.7%, whereas in OBO it is around 9.7%. Moreover, in the PO there are more SFAs, as it has a significantly higher PUFA:SFA ratio. This information provides additional evidence that OBO might be a more beneficial substitute for PO. A ratio of ω-6:ω-3 fatty acids ranging from 1:1 to 5:1 is considered the most favorable for human health. In recent years, however, this ratio has increased from 1:1 to 20:1 in the Western diet, compared to 45:1 in the South Asian diet [14]. It is observed that in OBO there is an increase in the ω-6:ω-3 ratio of about 36.77% while in PO it is 58.11%. In addition, the COX value of OBO is significantly higher (*p* < 0.05) than PO, confirming that the latter is more stable [46]. However, the higher the COX value, the healthier the oil, so it is once again indicated that OBO is a healthier alternative to PO [46]. Furthermore, it has been observed that there is typically an inverse link between the PUFA:SFA ratio and the COX value in natural oils, as well as their oxidative stability [47]. To evaluate the potential impact of fatty acids on cardiovascular disease, IA and IT are two of the most popular, trustworthy, and extensively utilized indices for all types of lipids and oils. While the thrombogenic index indicates an affinity for blood clot development in the arteries and cardiovascular disease, the atherogenic index reveals an early sign of accelerated atherosclerosis and can bolster our understanding of the inflammatory mechanisms linked to it [48]. PO exhibits significantly ~9 and ~10-fold higher IA and IT values compared to OBO, respectively. This finding is related to the fatty acid composition, providing additional evidence that PO is detrimental to human health. In addition, our results regarding IA and IT in corn, palm, and rapeseed oils are in line with the existing literature [48]. Similarly, the hypocholesterolemic/hypercholesterolemic ratio demonstrates that OBO significantly improves low-density lipoprotein levels by ~10-fold compared to high-density lipoprotein. Finally, the mathematical formulae of the health-promoting index and the hypocholesterolemic/hypercholesterolemic ratio have similarities, causing certain values to be identical in both indices. Nevertheless, it is evident that both ratios reduce when the samples are heated resulting from the degradation of the double bonds in fatty acids accordingly. No threshold has been established for HPI; however, a higher HPI indicates vast benefits for human health. As such, it was previously documented that dairy products (i.e., cheese, butter, milk, and yogurt) had an HPI range of 0.16–0.68 [49].

### 3.4. Evaluation of Primary and Secondary Oxidation By-Products

#### 3.4.1. Spectrophotometric Determination of Oxidation By-Products

Established assays were implemented to evaluate oil oxidation in terms of both primary and secondary oxidation by-products, the results of which are shown in Table 5. In the early phases of lipid oxidation, peroxides and hydroperoxides predominate; a key indicator for their evaluation is the peroxide value. The generation rate of hydroperoxides and the increase in PV are both affected by the oxidative stability of the seed oil [50]. The OBO and PO sample PV values range from 2.12–39.95 and 2.88–18.56 mmol H_2_O_2_/kg oil, respectively. Although OBO has a higher concentration of bioactive components (i.e., tocopherols and carotenoids), PO seems to be more stable during thermal oxidation. This could eventually be associated with fatty acid composition, as the OBO sample has plenty of UFAs which are susceptible to oxidation. Despite being a helpful biomarker in the initial phases of lipid oxidation, PV is ineffective for determining the level of oil stability at high temperatures. The off-flavor in oxidized edible oils is caused by the degradation of peroxides into secondary oxidation products, including aldehydes, ketones, and alcohols, as a result of their instability [51]. *p*-AV is regarded as a more dependable indicator of severe oxidative rancidity in oils since hydroperoxides are more thermolabile compounds than aldehydes [52]. The TBARS assay is also a selective assay that is used to identify secondary oxidation by-products. Malondialdehyde is an aldehyde generated during UFA degradation and produces a distinctive Schiff base compound that has a pink hue when the reaction is conducted under heat [53]. Despite OBO having a substantially higher total tocopherol and total carotenoid content than the PO sample, there were notable differences in PV between the two samples, suggesting that the oxidative stability was different. The same trend was observed in the assays regarding the secondary oxidation by-products. After 4 h of accelerated oxidation, TBA had a greater increase in the OBO sample (~335%) compared to PO (~72%), whereas a *p*-AV increase had a similar increase (~435% compared to ~44%). Similar to PV, the two latter assays revealed that degradation into secondary products caused peroxide formation to slow down in the late stages of oxidation, as also stated elsewhere [54]. Consequently, TV in both samples rose during the accelerated oxidation process and followed a pattern comparable to that of PV, indicating that the primary rather than secondary oxidation product evolution dictated the pattern of TV increase. Finally, CD values between the two samples were in line with the previous findings, as the OBO sample had a ~183% increase compared to ~29% from the PO sample after 4 h of thermal exposure. Interestingly though, PO had a higher CT value than OBO in each measurement, but negligible changes in its concentration were observed over time. All of these findings lead us to the conclusion that the high oxidation by-product concentration in the OBO sample was a matter of the high proportion of PUFAs. Fatty acids have a vast impact on the formation of oxidation by-products which cannot be mitigated by the presence of other antioxidant compounds.

#### 3.4.2. Chromatographic Determination of Volatile Compounds after Accelerated Oxidation

The volatile compounds formed by the accelerated oxidation process were measured for conductivity using the Rancimat method. All of these volatile compounds were transferred into measuring vessels that hold 60 mL of deionized water. Using HS-SPME/GC-MS, the volatile compounds in each water sample were examined. The sum of volatile aldehydes and ketones found in the Rancimat water samples during the oxidative stability test of the oils is also shown in Table 5. The rearrangement and scission of alkoxyl radicals is a potential process for the creation of these volatile compounds, particularly aldehydes, ketones, and alcohols [55]. The results showed that the OBO sample had a three-fold higher concentration of volatile odorous compounds than the PO samples, lining with the previous results regarding secondary oxidation products. Zhou et al. [56] found a similar association between *p*-AV and aldehyde compounds in walnut oil under the oxidation process. As a result, aldehydes are reliable indicators of the oxidative breakdown of seed oils. Major aldehydes for the OBO samples included *trans*, *trans*-2,4-heptadienal, *trans*-2-heptenal, hexanal, *trans*, *trans*-2,4-decadienal, and *trans*-2-pentenal and major ketones included *trans*-3-nonen-2-one, 2-cyclohexen-1-one, 3,5-octadien-2-one, and 4-hexen-2-one. Alternatively, the main aldehydes in the PO samples were hexanal, *trans*-2-heptenal, *trans*, *trans*-2,4-heptadienal, nonanal, and octanal, while the main ketones were 6-methyl-5-hepten-2-one and 5-pentyl-3-*H*-furan-2-one. Except for aldehydes and ketones, as well as other compounds like alcohols, carboxylic acids, alkanes, alkenes, and ethers, were found during the analysis of volatile compounds. In contrast, the samples of PO contain a variety of terpenes and terpenoids that contribute to their distinct flavor and aroma. Samples of PO contain specific terpenes such as linalool, terpinen-4-ol, eucalyptol, *D*-limonene, and camphor as well as terpenoids like *D*-carvone, *cis*-*p*-menthan-3-one, and dihydrocarvone. In conclusion, terpenes and terpenoids are vital parts of PO, offering both sensory qualities and possible health advantages [57].

### 3.5. ATR–FTIR Spectra Analysis

Currently, FTIR spectroscopy is being employed frequently in food research, specifically as a highly effective analytical technique for analyzing edible oils and fats [58]. It is a rapid method for identifying the molecular structure by associating each absorbance band with a particular functional group. FTIR serves as a vital tool for monitoring and controlling the quality of the production process, while also emphasizing evaluating the final oil products from manufacturers. Furthermore, the FTIR technique can be a practical method for detecting fats and oils in various stages of circulation, such as storage, distribution, and consumption [59].

**Figure 3:**
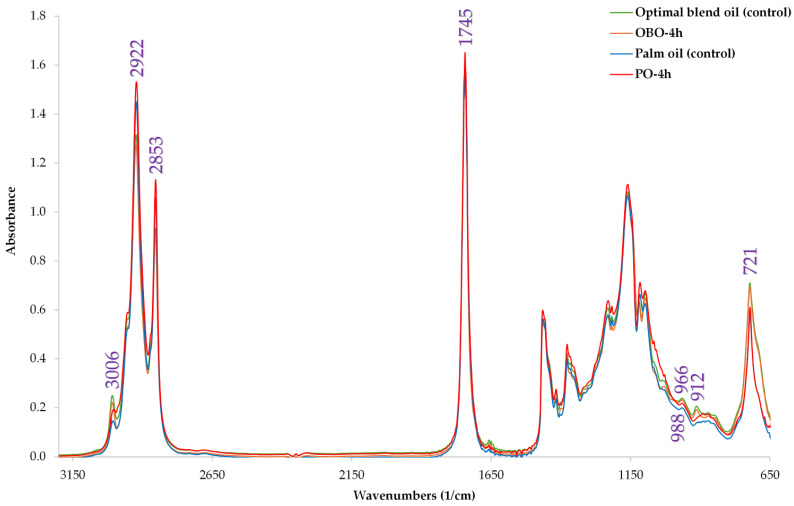
Stacked ATR–FTIR spectra of oil samples. Purple values indicate the major wavenumbers in specific functional groups.

The spectra in Figure 3 illustrate the characteristic peaks of the fatty acids in the oil samples at several oxidation stages, while in Table 6 the exact absorbance of each band is denoted. Major bands shown in the Figure could be attributed to previously discussed studies [60,61]. It can be seen that oil samples display slight variations in the position and absorbance of the bands as a result of their distinctive triglyceride composition, as per de la Mata et al. [62], who investigated oil blends with extra virgin olive oil (varietal and monovarietal) and several seed oils, including rapeseed, corn, flaxseed, peanut, safflower, sesame, soybean, grape seed, and high oleic sunflower oils. Firstly, it can be seen that from 2853 to 2922 cm^−1^ are the characteristic symmetric and non-symmetric C–H bond vibrations of the aliphatic part of the fatty acids. On the other hand, however, at 3006 cm^−1^ the stretching symmetric vibration of the *cis*-double bonds is distinguishable, whereas in the optimal sample, the absorbance is higher, demonstrating the existence of more *cis*-double bonds, due to the presence of more UFAs. It can be seen in Table 4 that the OBO has more UFAs than the PO after 4 h of oxidation. This is a reasonable result as the corn oil and the rapeseed oil possess more UFAs than the PO. This result is also supported by the absorption bands at 912 and 721 cm^−1^, where rocking and bending vibrations of –HC=CH– are highlighted, with the absorptions being higher in the optimal sample by up to 27% compared to PO. The weak stretching vibration at 1656 cm^−1^ corresponds to the C–C functional groups of PUFAs, as reported by Rozali et al. [63]. Finally, a band in 1745 cm^−1^ was observed in similar absorbances in all oil samples which indicates the presence of an ester carbonyl functional group.

### 3.6. Correlation Analyses

#### 3.6.1. Multiple Factor Analysis (MFA)

**Figure 4:**
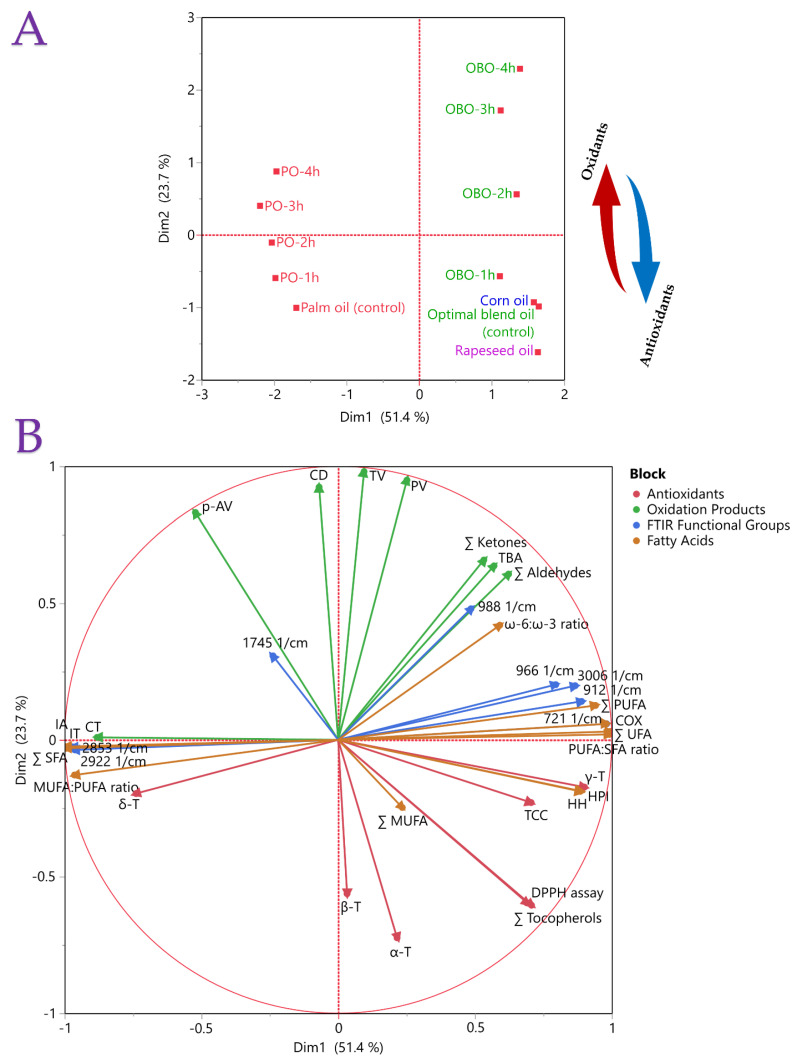
Plot (**A**) presents the results of multiple factor analysis for the measured samples, while Plot (**B**) shows the parameters in blocks between the samples of palm oil and the optimal blend oil.

It is feasible to compare the variables after they are transformed into orthogonal factors. According to the evaluations of the variable, these elements demonstrate how things are similar and different. Figure 4 displays the results of our implementation of MFA to examine the relationships between the measured variables. The total variance explained by these two dimensions was 51.4% and 23.7%, respectively. Figure 4A shows the factor scores for the first two dimensions for each measurement variable. The plot displays blocks of familiar elements because of their connection in the factor space. There was not a significant variation in the placement of pure oil samples compared to OBO; the samples have also been discriminated based on their antioxidant status. The pure substrate oil samples were positioned close to the OBO sample, as the blend consisted of these oils. The samples were also classified based on their antioxidant status and oxidative stability as shown by the respective arrows. For example, OBO was richer in antioxidant activity but poorer in oxidative stability than PO, as also indicated by the plot. A similar result in discriminant analysis was observed in a study by Kmiecik et al. [64]. They investigated how vegetable oils such as rapeseed oil, camelina oil, hemp oil, and linseed oil with various tocopherol content were blended to form a 5:1 ratio of ω-6/ω-3 fatty acids at different storage temperatures (control, 170 and 200 °C). Concerning their oxidative stability, the results showed that small subgroups of the same samples were in proximity, while they differed depending on the bioactive substance composition. In addition, a study from Cichocki et al. [65] employed blends of refined oil (rice bran oil) and cold-pressed oils including black cumin oil, rapeseed oil, pumpkin seed oil, milk thistle seed oil, hemp oil, black cumin oil, and linseed oil in different ratios. The authors satisfactorily discriminated total polar compounds and polymerized triacylglycerols between samples that were heated at 170 and 200 °C. Finally, this statistical tool could be employed when studying a single oil. Kmiecik et al. [66] used this approach to discriminate phytosterols and oxidation products from pressed, refined, and partially hydrogenated rapeseed oil during heating at 170 °C.

Figure 4B shows a measure of the factor that each set of variables contributes to each dimension. The diagram displays discrete positive and negative correlations between parameters. It was revealed that the sum of tocopherols and MUFA were positively correlated with dimension 1 (51.4%), whereas variables such as *p*-AV and CT were strongly negatively correlated with this dimension. For instance, a positive correlation was found between the parameters 988 cm^−1^ and *secondary* oxidation products, such as total ketones, aldehydes, and TBA value, as confirmed below. Therefore, samples with high levels of *trans*-fatty acids produced a greater amount of these oxidation by-products. Conversely, it was evident that the parameter 912 cm^−1^ showed a positive correlation with total PUFAs, as anticipated. A further interesting finding was the inverse relationship between the *p*-AV parameter and total tocopherols, suggesting that an elevation in tocopherol concentration led to a reduction in the *p*-AV value.

#### 3.6.2. Consensus Map

**Figure 5:**
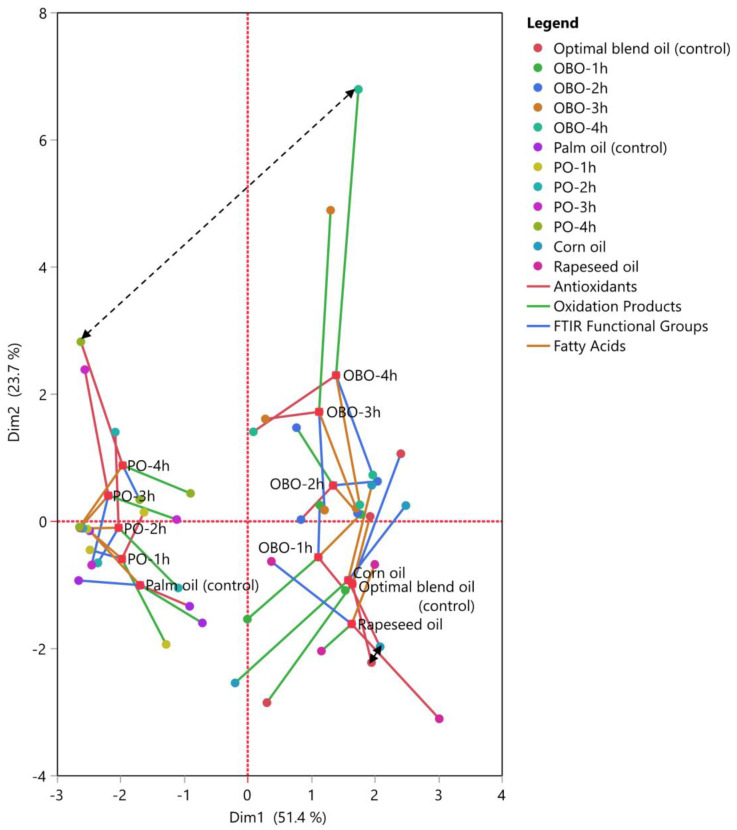
Consensus map between the optimal blend oil and the palm oil samples for the measured parameters in blocks. An example of high and low inertia between variables is highlighted with the black-dotted arrows.

To enhance data processing and more clearly interpret the correlation between the oil samples and variables, a consensus map was also employed and illustrated in Figure 5. In contrast to MFA, the consensus map displays both the average and individual sample responses side by side. All of the variables mentioned above (i.e., oil samples, bioactive compounds, oxidants, and functional groups) are graphically represented in this chart. It could also show how the samples varied or were similar concerning various parameters. The results were interpreted based on “inertia” values. Inertia is a measure of cluster coherence, which is another name for the within-cluster sum of squares. Data points within a cluster with a high inertia value are highly dissimilar to one another [67]. For example, the antioxidant variables of OBO at 0 h and corn oil showed low inertia, indicating that they had similarities in their total antioxidant activity. This finding was expected since the OBO sample mostly consisted of corn oil. On the other hand, oxidants from OBO at 4 h were positioned far from the corresponding oxidants from PO at 4 h showing large inertia, as their oxidation by-products were previously revealed to differ significantly (*p* < 0.05). To shed more light on the evaluation of inertia, the mentioned variables were highlighted with black-dotted arrows. However, it should be noted that each oil sample along with its parameters were placed in proximity and were discriminated from the other oil and its parameters. In conclusion, the correlation between the samples and the various variables is thoroughly illustrated by this graph. This approach enables the rapid classification and discrimination of samples while evaluating all variables.

#### 3.6.3. Multivariate Correlation Analysis (MCA)

**Figure 6:**
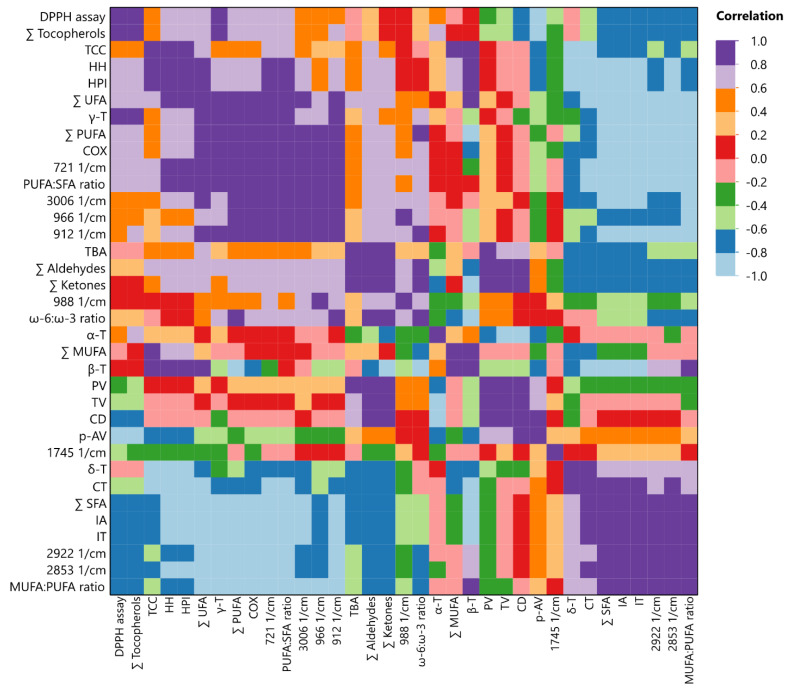
Analyzing measured variables through multivariate correlation.

To gain even more in-depth insight and understanding of the correlation among the variables, MCA was also employed. The main advantage of the specific analysis over the previous two is that the degree of positive or negative correlation between the variables can be measured. Figure 6 illustrates the results from this correlation analysis. The legend uses a color scale to represent positive or negative correlation values ranging from –1 to 1, as indicated by the following caption. To start with, it was observed that from all bioactive compounds (i.e., tocopherols, carotenoids, and UFAs), only γ-tocopherol had a strong positive (>0.8) correlation with DPPH^•^ inhibition activity. In our case, the higher antioxidant capacity from the OBO sample could be attributed to the two-fold higher concentration in the specific homolog than the PO sample. Interestingly, both δ-tocopherol and total SFAs were strongly positive correlated with increased CT values, as was observed in PO samples. Finally, a straightforward relation regarding secondary volatile by-products was observed, as TBA value was strongly correlated with total aldehydes and ketones rather than *p*-AV. This finding was also previously revealed through MFA.

## 4. Conclusions

In light of growing global awareness of the environment and the importance of a nutritious diet, there is an urgent need to develop oils that include enhanced nutritional properties. Due to its high content of SFAs, palm oil cannot meet this demand. Indeed, its high content of SFA has the potential to cause significant health issues for consumers. A novel blend of conventional vegetable oils consisting of corn oil and rapeseed oil in a ratio of 4:3 *w*/*w* was formulated and subjected to a series of tests. These assays included Rancimat accelerated oxidation conditions, an assessment of antioxidant inhibition, the measurement of oxidation by-products, and an evaluation of bioactive compounds (i.e., tocopherols, carotenoids, and UFAs). The results of this blended oil sample were then compared with those of palm oil. Palm oil had superior thermal stability compared to the optimal blended oil due to its high SFA content. On the other hand, the OBO sample contains a significant amount of oxidation by-products, which can be explained by the high proportion of PUFAs. Fatty acids exert a significant influence on the generation of oxidation by-products, and this influence cannot be alleviated by the presence of other antioxidant compounds. However, the blended oil exhibited excellent nutritional quality as its antioxidant capacity was ~33% higher than palm oil. ATR-FTIR spectra also confirmed the presence of a significant amount of SFAs in palm oil and UFAs in the blended oil, indicating that the blended oil is more suitable for ingestion. This study has the potential to serve as a foundation for the large-scale production of oil blends that are high in antioxidants and nutrients. These blends can be prepared using common vegetable oils that also exhibit significant resistance to oxidation and could reduce palm oil in the food industry. Moreover, it is preferable to blend oils as they mutually boost each other. For instance, rapeseed oil possesses a greater proportion of MUFAs compared to corn oil, whereas corn oil has a greater concentration of tocopherols than rapeseed oil. Their combination thus incorporates optimized and increased characteristics obtained from both oils.

## Figures and Tables

**Table 1:** The Main Effects Screening design was used to optimize the optimal blend of vegetable oils using the actual and coded levels of the independent variables.

Independent Variables	Code Units	Coded Variable Level				
		1	2	3	4	5
Palm oil (PO)	*X*_1_	0	1	2	3	4
Corn oil (CO)	*X*_2_	0	1	2	3	4
Rapeseed oil (RSO)	*X*_3_	0	1	2	3	4
Sunflower oil (SO)	*X*_4_	0	1	2	3	4

**Table 2:** The experimental results for the four independent variables under investigation, as well as the dependent variable response in terms of percentages of oxidative stability (%OS) by using the DPPH method and percentages of oxidative power (%OP) by using conjugated diene (CD) and conjugated triene (CT) after two hours of storage at boiling water. Additionally, pure substrate oils (20–23) are presented.

Design Point	*X*_1_ (PO)	*X*_2_ (CO)	*X*_3_ (RSO)	*X*_4_ (SO)	DPPH ^1^	DPPH-2h	%OS ^2^	CD ^3^	CD-2h	%OP-CD ^2^	CT ^3^	CT-2h	%OP-CT ^2^
1	0 (0)	1 (33.3)	0 (0)	2 (66.7)	199.66 ± 10.18	156.43 ± 5.48	78.35	12.10 ± 0.37	14.14 ± 0.40	16.87	6.71 ± 0.48	6.88 ± 0.37	2.58
2	1 (14.3)	0 (0)	2 (28.6)	4 (57.1)	181.00 ± 7.42	138.87 ± 7.22	76.73	16.33 ± 1.06	16.47 ± 0.56	0.86	7.80 ± 0.51	7.87 ± 0.31	0.98
3	2 (25)	2 (25)	4 (50)	0 (0)	168.45 ± 11.79	153.83 ± 8.31	91.32	9.02 ± 0.31	13.52 ± 0.42	49.79	4.42 ± 0.27	4.88 ± 0.36	10.38
4	3 (30)	2 (20)	3 (30)	2 (20)	191.58 ± 9.58	137.82 ± 9.92	71.94	12.01 ± 0.65	18.23 ± 1.17	51.76	5.65 ± 0.21	7.23 ± 0.50	28.11
5	3 (37.5)	1 (12.5)	1 (12.5)	3 (37.5)	182.63 ± 4.57	145.75 ± 8.16	79.81	13.00 ± 0.94	17.85 ± 1.18	37.28	6.73 ± 0.38	7.46 ± 0.51	10.85
6	4 (36.4)	0 (0)	4 (36.4)	3 (27.3)	174.02 ± 3.48	130.87 ± 9.68	75.20	10.97 ± 0.63	19.94 ± 1.50	81.75	5.14 ± 0.35	7.43 ± 0.22	44.62
7	1 (12.5)	4 (50)	0 (0)	3 (37.5)	212.16 ± 9.55	16.55 ± 1.03	7.80	11.20 ± 0.72	21.51 ± 1.55	92.11	7.22 ± 0.30	9.56 ± 0.64	32.45
8	4 (50)	3 (37.5)	1 (12.5)	0 (0)	181.50 ± 6.17	151.49 ± 4.85	83.46	11.27 ± 0.28	14.92 ± 0.90	32.34	6.78 ± 0.42	7.13 ± 0.23	5.19
9	2 (16.7)	3 (25)	3 (25)	4 (33.3)	206.18 ± 10.72	157.16 ± 11.16	76.22	14.56 ± 0.51	14.71 ± 0.37	1.06	6.02 ± 0.13	6.11 ± 0.44	1.56
10	3 (20)	4 (26.7)	4 (26.7)	4 (26.7)	220.76 ± 16.12	147.22 ± 6.33	66.69	10.39 ± 0.49	14.42 ± 0.98	38.80	5.38 ± 0.11	5.74 ± 0.41	6.58
11	1 (11.1)	3 (33.3)	4 (44.4)	1 (11.1)	228.98 ± 7.56	163.84 ± 4.75	71.55	7.13 ± 0.41	13.81 ± 0.98	93.50	4.00 ± 0.10	4.73 ± 0.13	18.34
12	2 (33.3)	1 (16.7)	2 (33.3)	1 (16.7)	173.82 ± 10.08	145.36 ± 7.12	83.62	9.30 ± 0.26	14.30 ± 0.59	53.73	4.88 ± 0.33	5.52 ± 0.34	13.15
13	4 (57.1)	2 (28.6)	0 (0)	1 (14.3)	181.18 ± 13.59	139.17 ± 3.48	76.81	11.79 ± 0.84	16.44 ± 0.94	39.43	7.38 ± 0.44	7.91 ± 0.16	7.26
14	1 (20)	1 (20)	3 (60)	0 (0)	177.21 ± 10.63	137.31 ± 9.20	77.49	7.31 ± 0.26	12.24 ± 0.61	67.49	3.19 ± 0.15	3.72 ± 0.21	16.70
15	0 (0)	3 (37.5)	2 (25)	3 (37.5)	194.19 ± 3.88	150.85 ± 6.49	77.68	11.06 ± 0.44	14.40 ± 0.56	30.24	5.54 ± 0.34	5.68 ± 0.16	2.69
16	0 (0)	2 (28.6)	1 (14.3)	4 (57.1)	192.49 ± 14.05	144.70 ± 10.27	75.17	12.41 ± 0.81	15.31 ± 0.90	23.35	6.63 ± 0.14	6.74 ± 0.47	1.72
17	2 (40)	0 (0)	1 (20)	2 (40)	174.13 ± 7.31	126.68 ± 3.93	72.75	11.59 ± 0.45	15.17 ± 0.59	30.84	6.35 ± 0.33	6.62 ± 0.21	4.26
18	0 (0)	4 (50)	3 (37.5)	1 (12.5)	203.01 ± 14.21	160.44 ± 3.69	79.03	9.44 ± 0.69	13.33 ± 0.52	41.22	4.65 ± 0.12	4.93 ± 0.10	5.92
19	4 (33.3)	4 (33.3)	2 (16.7)	2 (16.7)	198.10 ± 7.73	143.92 ± 8.78	72.65	10.39 ± 0.25	14.74 ± 0.55	41.84	6.33 ± 0.29	6.54 ± 0.32	3.28
20	3 (100)	0 (0)	0 (0)	0 (0)	162.66 ± 5.86	117.26 ± 3.75	72.09	13.14 ± 0.66	16.67 ± 1.25	26.88	8.59 ± 0.64	8.91 ± 0.20	3.76
21	0 (0)	3 (100)	0 (0)	0 (0)	243.76 ± 15.11	185.21 ± 5.37	75.98	10.19 ± 0.49	13.51 ± 0.34	32.56	6.93 ± 0.36	7.00 ± 0.52	1.04
22	0 (0)	0 (0)	3 (100)	0 (0)	171.56 ± 4.12	134.00 ± 4.69	78.11	14.43 ± 0.43	39.72 ± 2.46	175.20	1.89 ± 0.08	9.03 ± 0.51	376.73
23	0 (0)	0 (0)	0 (0)	3 (100)	187.22 ± 6.74	21.13 ± 1.29	11.29	12.31 ± 0.37	16.70 ± 0.97	35.60	6.58 ± 0.16	7.58 ± 0.42	15.30

Weighed quantities (in g) of oils in a total sum of 100 g are presented in parentheses in the Coded Variables Levels. Values are expressed as the mean values of triplicates (±standard deviation). ^1^ 2,2-Diphenyl-1-picrylhydrazyl (DPPH) assay in μmol TEAC/kg oil; ^2^ The %OS and %OP represent the increase in oxidative stability and oxidation power, respectively, from the initial values; ^3^ conjugated dienes (CD) and conjugated trienes (CT) in mmol/kg oil; PO: palm oil; CO: corn oil; RSO: rapeseed oil; and SO: sunflower oil.

**Table 3:** Antioxidant parameters between the samples of palm oil and the optimal blend oil.

Oil Samples	DPPH ^1^	TCC ^2^	α-T ^3^	β-T ^3^	γ-T ^3^	δ-T ^3^	∑ Tocopherols ^4^
Optimal blend oil (control)	149.08 ± 5.67 ^c^	2.80 ± 0.11 ^b^	247.62 ± 6.93 ^b^	31.26 ± 1.03 ^a,b^	387.04 ± 15.48 ^b^	25.43 ± 1.14 ^d,e^	691.34 ± 24.59 ^b^
OBO-1 h	139.10 ± 3.76 ^c^	2.66 ± 0.18 ^b^	187.23 ± 8.43 ^c^	29.79 ± 1.01 ^b,c^	363.65 ± 17.09 ^b,c^	24.00 ± 1.46 ^e^	604.67 ± 27.99 ^c^
OBO-2 h	115.81 ± 4.98 ^d^	2.51 ± 0.14 ^b,c^	79.80 ± 1.84 ^d^	31.16 ± 0.69 ^a,b^	337.91 ± 7.43 ^c,d^	23.20 ± 1.42 ^e^	472.07 ± 11.37 ^d,e^
OBO-3 h	83.72 ± 2.01 ^e^	2.22 ± 0.05 ^c,d^	31.47 ± 1.61 ^e^	27.71 ± 1.97 ^b,c^	326.16 ± 14.35 ^c,d,e^	22.64 ± 1.27 ^e^	407.99 ± 19.19 ^e,f^
OBO-4 h	73.55 ± 3.68 ^e,f^	1.95 ± 0.13 ^d^	nd *	26.08 ± 1.88 ^c^	301.23 ± 19.88 ^d,e^	22.18 ± 0.67 ^e^	349.49 ± 22.42 ^f^
Palm oil (control)	111.92 ± 3.47 ^d^	0.58 ± 0.02 ^e^	312.41 ± 23.43 ^a^	nd	174.2 ± 9.41 ^f^	40.37 ± 2.02 ^a^	526.98 ± 34.86 ^d^
PO-1 h	75.08 ± 5.41 ^e,f^	0.57 ± 0.04 ^e^	204.73 ± 12.69 ^c^	nd	156.24 ± 9.69 ^f^	39.53 ± 1.94 ^a,b^	400.49 ± 24.32 ^f^
PO-2 h	59.60 ± 2.03 ^f^	0.56 ± 0.01 ^e^	105.07 ± 3.15 ^d^	nd	135.88 ± 9.65 ^f,g^	36.77 ± 0.92 ^a,b,c^	277.72 ± 13.72 ^g^
PO-3 h	40.78 ± 0.94 ^g^	0.43 ± 0.02 ^e^	43.98 ± 1.76 ^e^	nd	100.39 ± 3.51 ^g,h^	34.28 ± 2.26 ^c^	178.65 ± 7.54 ^h^
PO-4 h	30.33 ± 1.70 ^g^	0.31 ± 0.01 ^e^	37.79 ± 2.46 ^e^	nd	65.40 ± 2.42 ^h^	28.91 ± 1.76 ^d^	132.10 ± 6.64 ^h^
Corn oil	245.59 ± 15.96 ^a^	0.50 ± 0.01 ^e^	199.14 ± 7.37 ^c^	26.03 ± 1.43 ^c^	522.88 ± 21.96 ^a^	35.31 ± 2.30 ^b,c^	783.36 ± 33.06 ^a^
Rapeseed oil	171.56 ± 11.49 ^b^	5.62 ± 0.34 ^a^	254.54 ± 7.13 ^b^	35.42 ± 2.23 ^a^	297.21 ± 11.29 ^e^	13.85 ± 0.35 ^f^	601.03 ± 21.00 ^c^

Values are expressed as the mean values of triplicates (±standard deviation). Within each column, statistically significant differences (*p* < 0.05) are denoted by different superscript letters (e.g., a–h). nd *: not detected. ^1^ 2,2-Diphenyl-1-picrylhydrazyl (DPPH) assay in μmol TEAC/kg oil; ^2^ totalcarotenoid content (TCC) in mg lutein/kg oil; ^3^ α-, β-, γ-, and δ-tocopherols in mg/kg oil; ^4^ sum of α-, β-, γ-, and δ-tocopherols in mg/kg oil.

**Table 4:** Variations in the fatty acid percentages (%) between the optimal blend oil and the palm oil samples.

Oil Samples	∑ SFA ^1^	∑ MUFA ^2^	∑ PUFA ^3^	∑ UFA ^4^	PUFA: SFA Ratio	MUFA: PUFA Ratio	ω-6: ω-3 Ratio	COX ^5^	IA ^6^	IT ^7^	HH ^8^	HPI ^9^
Optimal blend oil (control)	9.22 ± 0.34 ^b,c^	43.62 ± 1.18 ^b^	47.16 ± 3.37 ^b^	90.78 ± 4.55 ^a^	5.11 ± 0.18 ^a^	0.93 ± 0.04 ^d^	39.16 ± 0.98 ^d^	5.43 ± 0.36 ^b^	0.10 ± 0 ^c^	0.18 ± 0 ^e^	10.35 ± 0.14 ^b^	10.36 ± 0.14 ^b^
OBO-1 h	10.00 ± 0.53 ^b,c^	42.80 ± 2.05 ^b^	47.20 ± 1.77 ^b^	90.00 ± 3.82 ^a^	4.72 ± 0.07 ^b^	0.91 ± 0.01 ^d^	39.52 ± 0.67 ^d^	5.42 ± 0.21 ^b^	0.11 ± 0 ^c^	0.20 ± 0 ^d,e^	9.36 ± 0.11 ^c^	9.36 ± 0.11 ^c^
OBO-2 h	10.35 ± 0.69 ^b,c^	42.65 ± 1.49 ^b^	47.00 ± 2.91 ^b^	89.65 ± 4.41 ^a^	4.54 ± 0.02 ^b,c^	0.91 ± 0.02 ^d^	40.75 ± 0.04 ^d^	5.39 ± 0.32 ^b^	0.11 ± 0 ^c^	0.21 ± 0 ^d,e^	9.02 ± 0.17 ^c,d^	9.02 ± 0.17 ^c,d^
OBO-3 h	10.85 ± 0.35 ^b,c^	41.64 ± 3.00 ^b^	47.51 ± 2.03 ^b^	89.15 ± 5.02 ^a^	4.38 ± 0.05 ^c,d^	0.88 ± 0.03 ^d^	47.63 ± 1.53 ^c^	5.42 ± 0.25 ^b^	0.12 ± 0 ^c^	0.22 ± 0.01 ^d,e^	8.43 ± 0.21 ^e^	8.43 ± 0.21 ^e^
OBO-4 h	11.23 ± 0.53 ^b,c^	40.69 ± 1.59 ^b^	48.08 ± 3.08 ^b^	88.77 ± 4.66 ^a^	4.28 ± 0.07 ^d^	0.85 ± 0.02 ^d^	53.56 ± 0.05 ^b^	5.46 ± 0.34 ^b^	0.11 ± 0 ^c^	0.23 ± 0 ^d,e^	8.76 ± 0.06 ^d,e^	8.76 ± 0.06 ^d,e^
Palm oil (control)	49.30 ± 2.31 ^a^	40.21 ± 0.88 ^b^	10.49 ± 0.39 ^d^	50.70 ± 1.27 ^b^	0.21 ± 0 ^e^	3.83 ± 0.06 ^b^	14.23 ± 0.20 ^g^	1.56 ± 0.05 ^c^	0.94 ± 0.02 ^b^	1.80 ± 0.04 ^c^	1.13 ± 0.02 ^g^	1.07 ± 0.02 ^g^
PO-1 h	49.63 ± 3.33 ^a^	40.40 ± 1.54 ^b^	9.97 ± 0.53 ^d^	50.37 ± 2.07 ^b^	0.20 ± 0 ^e^	4.05 ± 0.06 ^a^	18.99 ± 0.44 ^f^	1.49 ± 0.07 ^c^	0.94 ± 0.02 ^b^	1.86 ± 0.05 ^b,c^	1.12 ± 0.03 ^g^	1.07 ± 0.03 ^g^
PO-2 h	50.53 ± 2.53 ^a^	39.69 ± 2.78 ^b^	9.79 ± 0.54 ^d^	49.47 ± 3.32 ^b^	0.19 ± 0 ^e^	4.05 ± 0.06 ^a^	19.67 ± 0.69 ^f^	1.46 ± 0.08 ^c^	0.98 ± 0.01 ^a,b^	1.93 ± 0.03 ^a,b^	1.08 ± 0.02 ^g^	1.02 ± 0.01 ^g^
PO-3 h	51.06 ± 2.71 ^a^	39.23 ± 1.02 ^b^	9.71 ± 0.35 ^d^	48.94 ± 1.37 ^b^	0.19 ± 0 ^e^	4.04 ± 0.04 ^a^	19.90 ± 0.16 ^f^	1.44 ± 0.05 ^c^	1.01 ± 0.03 ^a^	1.97 ± 0.05 ^a^	1.04 ± 0.03 ^g^	0.99 ± 0.03 ^g^
PO-4 h	51.14 ± 2.49 ^a^	39.34 ± 1.14 ^b^	9.51 ± 0.39 ^d^	48.86 ± 1.53 ^b^	0.19 ± 0 ^e^	4.14 ± 0.05 ^a^	22.50 ± 0.66 ^e^	1.42 ± 0.05 ^c^	1.02 ± 0.02 ^a^	1.99 ± 0.03 ^a^	1.04 ± 0.02 ^g^	0.99 ± 0.02 ^g^
Corn oil	12.71 ± 0.72 ^b^	27.59 ± 2.01 ^c^	59.69 ± 2.71 ^a^	87.29 ± 4.72 ^a^	4.70 ± 0.05 ^b^	0.46 ± 0.01 ^e^	64.80 ± 1.69 ^a^	6.53 ± 0.31 ^a^	0.13 ± 0 ^c^	0.27 ± 0 ^d^	7.59 ± 0.03 ^f^	7.59 ± 0.03 ^f^
Rapeseed oil	7.15 ± 0.27 ^c^	61.99 ± 2.85 ^a^	30.86 ± 1.87 ^c^	92.85 ± 4.72 ^a^	4.31 ± 0.1 ^d^	2.01 ± 0.03 ^c^	2.41 ± 0.03 ^h^	4.82 ± 0.29 ^b^	0.05 ± 0 ^d^	0.10 ± 0 ^f^	18.33 ± 0.31 ^a^	18.33 ± 0.31 ^a^

Values are expressed as the mean values of triplicates (±standard deviation). Within each column, statistically significant differences (*p* < 0.05) are denoted by different superscript letters (e.g., a–h). ^1^ SFAs, saturated fatty acids (%): Sum of C12:0, lauric acid; C14:0, myristic acid; C16:0, palmitic acid; C18:0, stearic acid; C20:0, arachidic acid; and C22:0, behenic acid. ^2^ MUFAs, monounsaturated fatty acids (%): Sum of C16:1, palmitoleic acid; and C18:1, oleic acid. ^3^ PUFAs, polyunsaturated fatty acids (%): Sum of C18:2, ω-6, linoleic acid and C18:3, ω-3, linolenic acid. ^4^ UFAs, unsaturated fatty acids (%): Sum of MUFAs and PUFAs. ^5^ COX, calculated oxidizability value. ^6^ IA, Index of atherogenicity. ^7^ IT, Index of thrombogenicity. ^8^ HH, hypocholesterolemic/hypercholesterolemic ratio. ^9^ HPI, health-promoting index.

**Table 5:** Oxidant parameters between the samples of palm oil and the optimal blend oil.

Oil Samples	PV ^1^	*p*-AV ^2^	TV ^3^	CD ^4^	CT ^4^	TBA ^5^	∑ Aldehydes ^6^	∑ Ketones ^6^
Optimal blend oil (control)	2.12 ± 0.09 ^j^	4.34 ± 0.13 ^h^	8.58 ± 0.31 ^h^	11.44 ± 0.85 ^g,h^	4.27 ± 0.29 ^c^	2.04 ± 0.07 ^e,f^	–	–
OBO-1 h	10.53 ± 0.35 ^e,f^	8.10 ± 0.47 ^f^	29.16 ± 1.16 ^e^	17.57 ± 1.04 ^d,e,f^	4.74 ± 0.14 ^c^	4.08 ± 0.21 ^d^	161.41 ± 11.14 ^d,e^	7.65 ± 0.18 ^f^
OBO-2 h	21.78 ± 1.05 ^c^	13.24 ± 0.58 ^e^	56.80 ± 2.67 ^c^	21.90 ± 0.68 ^c^	4.25 ± 0.10 ^c^	6.14 ± 0.17 ^c^	356.82 ± 7.85 ^c^	14.17 ± 0.78 ^c^
OBO-3 h	36.44 ± 2.44 ^b^	18.28 ± 0.49 ^b,c^	91.16 ± 5.38 ^b^	29.62 ± 1.69 ^a^	4.52 ± 0.20 ^c^	8.00 ± 0.38 ^b^	426.70 ± 14.08 ^b^	24.69 ± 1.80 ^b^
OBO-4 h	39.95 ± 0.96 ^a^	23.23 ± 0.46 ^a^	103.13 ± 2.38 ^a^	32.39 ± 2.07 ^a^	4.48 ± 0.28 ^c^	8.88 ± 0.65 ^a^	574.75 ± 20.12 ^a^	31.40 ± 1.41 ^a^
Palm oil (control)	2.88 ± 0.20 ^i,j^	13.67 ± 0.86 ^e^	19.44 ± 1.27 ^f^	15.78 ± 0.65 ^f^	8.35 ± 0.37 ^a^	1.49 ± 0.07 ^f^	–	–
PO-1 h	5.33 ± 0.40 ^h,i^	16.31 ± 0.57 ^d^	26.96 ± 1.37 ^e^	16.85 ± 0.93 ^e,f^	8.14 ± 0.43 ^a^	1.87 ± 0.12 ^e,f^	115.58 ± 6.70 ^f^	3.31 ± 0.10 ^g^
PO-2 h	8.32 ± 0.52 ^f,g^	16.78 ± 0.87 ^c,d^	33.42 ± 1.90 ^e^	19.48 ± 1.34 ^c,d,e^	8.31 ± 0.47 ^a^	2.25 ± 0.11 ^e,f^	132.81 ± 8.63 ^e,f^	8.18 ± 0.27 ^e,f^
PO-3 h	12.80 ± 0.51 ^e^	17.91 ± 0.73 ^c,d^	43.52 ± 1.76 ^d^	25.52 ± 1.38 ^b^	8.63 ± 0.49 ^a^	2.21 ± 0.05 ^e,f^	133.83 ± 6.96 ^d,e,f^	9.60 ± 0.32 ^d,e^
PO-4 h	18.56 ± 0.93 ^d^	19.67 ± 0.51 ^b^	56.78 ± 2.37 ^c^	20.34 ± 1.28 ^c,d^	8.20 ± 0.29 ^a^	2.57 ± 0.07 ^e^	165.02 ± 4.62 ^d^	10.46 ± 0.58 ^d^
Corn oil	5.66 ± 0.29 ^g,h^	6.28 ± 0.42 ^g^	17.60 ± 1.00 ^f,g^	10.19 ± 0.31 ^h^	6.93 ± 0.35 ^b^	1.48 ± 0.09 ^f^	–	–
Rapeseed oil	5.07 ± 0.38 ^h,i^	1.41 ± 0.05 ^i^	11.55 ± 0.81 ^g,h^	14.43 ± 0.40 ^f,g^	1.89 ± 0.14 ^d^	6.05 ± 0.39 ^c^	–	–

Values are expressed as the mean values of triplicates (±standard deviation). Within each column, statistically significant differences (*p* < 0.05) are denoted by different superscript letters (e.g., a–j). ^1^ Peroxide value (PV) assay in mmol H_2_O_2_/kg oil; ^2^ *p*-anisidine value (*p*-AV) assay; ^3^ Totox value (TV); ^4^ conjugated dienes (CD) and conjugated trienes (CT) in mmol/kg oil; ^5^ thiobarbituric acid (TBA) assay in mmol malondialdehyde equivalents (MDAE)/kg oil; and ^6^ Sum of volatile compounds of aldehydes and ketones in μg 2-octanol equivalents/kg oil.

**Table 6:** ATR–FTIR functional groups between the samples of palm oil and the optimal blend oil.

Oil Samples	Wavenumbers (1/cm)							
	3006	2922	2853	1745	988	966	912	721
Optimal blend oil (control)	0.249 ± 0.005 ^a^	1.314 ± 0.05 ^a,b,c,d,e^	0.933 ± 0.024 ^b,c^	1.545 ± 0.100 ^a^	0.229 ± 0.016 ^a^	0.239 ± 0.012 ^a^	0.206 ± 0.015 ^a^	0.709 ± 0.040 ^a^
OBO-1 h	0.212 ± 0.011 ^b,c,d^	1.265 ± 0.081 ^d,e^	0.899 ± 0.032 ^c^	1.553 ± 0.059 ^a^	0.211 ± 0.006 ^a^	0.220 ± 0.011 ^a,b,c,d^	0.184 ± 0.007 ^a,b,c^	0.692 ± 0.017 ^a^
OBO-2 h	0.226 ± 0.006 ^a,b^	1.276 ± 0.074 ^c,d,e^	0.915 ± 0.049 ^c^	1.500 ± 0.109 ^a^	0.226 ± 0.011 ^a^	0.232 ± 0.017 ^a,b,c^	0.195 ± 0.009 ^a,b^	0.701 ± 0.018 ^a^
OBO-3 h	0.212 ± 0.007 ^b,c,d^	1.276 ± 0.065 ^c,d,e^	0.905 ± 0.056 ^c^	1.497 ± 0.091 ^a^	0.217 ± 0.008 ^a^	0.221 ± 0.008 ^a,b,c,d^	0.182 ± 0.004 ^b,c^	0.682 ± 0.022 ^a,b^
OBO-4 h	0.220 ± 0.015 ^b,c^	1.271 ± 0.064 ^c,d,e^	0.908 ± 0.023 ^c^	1.509 ± 0.112 ^a^	0.229 ± 0.010 ^a^	0.232 ± 0.012 ^a,b,c,d^	0.192 ± 0.008 ^a,b^	0.694 ± 0.041 ^a^
Palm oil (control)	0.146 ± 0.006 ^g^	1.449 ± 0.080 ^a,b,c,d^	1.058 ± 0.060 ^a,b^	1.496 ± 0.087 ^a^	0.200 ± 0.011 ^a^	0.200 ± 0.005 ^d^	0.140 ± 0.004 ^e^	0.589 ± 0.041 ^c^
PO-1 h	0.147 ± 0.008 ^g^	1.497 ± 0.111 ^a,b^	1.088 ± 0.032 ^a^	1.524 ± 0.078 ^a^	0.211 ± 0.014 ^a^	0.208 ± 0.008 ^a,b,c,d^	0.145 ± 0.010 ^d,e^	0.594 ± 0.024 ^c^
PO-2 h	0.162 ± 0.009 ^f,g^	1.487 ± 0.088 ^a,b,c^	1.096 ± 0.027 ^a^	1.473 ± 0.078 ^a^	0.210 ± 0.010 ^a^	0.206 ± 0.014 ^b,c,d^	0.145 ± 0.004 ^d,e^	0.586 ± 0.021 ^c^
PO-3 h	0.173 ± 0.005 ^e,f^	1.472 ± 0.062 ^a,b,c,d^	1.084 ± 0.037 ^a^	1.494 ± 0.072 ^a^	0.206 ± 0.007 ^a^	0.201 ± 0.006 ^c,d^	0.142 ± 0.004 ^d,e^	0.579 ± 0.013 ^c^
PO-4 h	0.187 ± 0.010 ^d,e^	1.531 ± 0.070 ^a^	1.131 ± 0.055 ^a^	1.570 ± 0.115 ^a^	0.224 ± 0.015 ^a^	0.218 ± 0.007 ^a,b,c,d^	0.158 ± 0.005 ^d,e^	0.610 ± 0.015 ^b,c^
Corn oil	0.212 ± 0.006 ^b,c,d^	1.223 ± 0.055 ^e^	0.880 ± 0.058 ^c^	1.425 ± 0.097 ^a^	0.222 ± 0.012 ^a^	0.235 ± 0.014 ^a,b^	0.203 ± 0.005 ^a,b^	0.704 ± 0.015 ^a^
Rapeseed oil	0.199 ± 0.006 ^c,d^	1.296 ± 0.074 ^b,c,d,e^	0.922 ± 0.058 ^c^	1.427 ± 0.104 ^a^	0.204 ± 0.012 ^a^	0.212 ± 0.011 ^a,b,c,d^	0.164 ± 0.007 ^c,d^	0.680 ± 0.018 ^a,b^

Values are expressed as the mean values of triplicates (±standard deviation). Within each column, statistically significant differences (*p* < 0.05) are denoted by different superscript letters (e.g., a–g).

## Data Availability

All related data and methods are presented in this paper. Additional inquiries should be addressed to the corresponding author.
